# Hydrogen-Rich Water Ameliorates Total Body Irradiation-Induced Hematopoietic Stem Cell Injury by Reducing Hydroxyl Radical

**DOI:** 10.1155/2017/8241678

**Published:** 2017-01-22

**Authors:** Junling Zhang, Xiaolei Xue, Xiaodan Han, Yuan Li, Lu Lu, Deguan Li, Saijun Fan

**Affiliations:** Tianjin Key Laboratory of Radiation Medicine and Molecular Nuclear Medicine, Institute of Radiation Medicine, Peking Union Medical College and Chinese Academy of Medical Science, Tianjin 300192, China

## Abstract

We examined whether consumption of hydrogen-rich water (HW) could ameliorate hematopoietic stem cell (HSC) injury in mice with total body irradiation (TBI). The results indicated that HW alleviated TBI-induced HSC injury with respect to cell number alteration and to the self-renewal and differentiation of HSCs. HW specifically decreased hydroxyl radical (^∙^OH) levels in the c-kit^+^ cells of 4 Gy irradiated mice. Proliferative bone marrow cells (BMCs) increased and apoptotic c-kit^+^ cells decreased in irradiated mice uptaken with HW. In addition, the mean fluorescence intensity (MFI) of *γ*-H2AX and percentage of 8-oxoguanine positive cells significantly decreased in HW-treated c-kit^+^ cells, indicating that HW can alleviate TBI-induced DNA damage and oxidative DNA damage in c-kit^+^ cells. Finally, the cell cycle (P21), cell apoptosis (BCL-XL and BAK), and oxidative stress (NRF2, HO-1, NQO1, SOD, and GPX1) proteins were significantly altered by HW in irradiated mouse c-kit^+^ cells. Collectively, the present results suggest that HW protects against TBI-induced HSC injury.

## 1. Introduction

Total body irradiation (TBI) may induce injury in many tissues and organs. Direct action refers to the effects of TBI on bioactive macromolecules such as proteins and nucleic acids. Ionization, excitation, chemical bond rupture, and changes in molecular structure all occur during this process, leading to abnormal function and metabolic disorders. Indirect action occurs via the generation of free radicals by water radiolysis [1]. The hydroxyl radical (^∙^OH), the free radical component that is one of the strongest oxidant species and that reacts with nucleic acids, lipids, and proteins, accounts for approximately 75% of lethal DNA damage caused by ionizing radiation [2]. Therefore, high concentrations of ^∙^OH radical scavengers may be promising radioprotective agents.

It is well known that molecular hydrogen (H_2_) acts as an antioxidant by efficiently reducing ^∙^OH. Studies have shown that H_2_ plays an important role in animal disease models, such as focal ischemia and reperfusion in rats [3], depressive-like behavior, and obstructive jaundice in mice [4]. In addition, it has been reported that H_2_ protects mice from TBI-induced injury in many tissues and organs, including bone marrow cells [5–7], heart [8], testis [6, 7], intestine [5], lung [9], and skin [10–12]. In the present study, we explore the radioprotective effect of HW on the injury of hematopoietic stem cells (HSCs).

HSCs have the ability to replenish themselves and to differentiate into blood cells of all hematopoietic lineages. As one of the best-characterized somatic stem cell types among all tissue stem cells, HSCs reside in a specialized redox niche with low levels of oxygen and limit production of reactive oxygen species (ROS) [13]. C-kit, also named CD117, is a cytokine receptor expressed on the surface of hematopoietic stem cells. C-kit positive (c-kit^+^) cells are the subset of total HSCs and hematopoietic progenitor cells (HPCs). No mature hematopoietic cells markers, such as Ter119, B220, CD11b, Gr1, CD4, CD8, and IL3(lineage), are found on the surface of murine HSCs and HPCs, except c-kit and sca1. HSCs are identified as lineage^−^sca1^+^c-kit^+^(LSK) and HPCs are identified as lineage^−^sca1^−^c-kit^+^, respectively. Thus, c-kit^+^ cells may represent the total HSCs and HPCs.

The hematopoietic system is highly radiosensitive, and TBI may induce acute hematopoietic syndrome, residual bone marrow injury, and hematopoietic stem cell (HSC) senescence [14, 15]. The potential mechanisms involved in HSCs' injury caused by TBI include the induction of HSC apoptosis, differentiation, and senescence [1]. Thus, it is necessary to identify and characterize radioprotective agents that guard against TBI-induced hematopoietic system injury.

Our present results show that HW may elevate the survival rate of mice lethally irradiated. Moreover, the number of bone marrow cells (BMCs) and the rate of self-renewal and differentiation are all improved in irradiated mice under the condition of HW consumption. HW significantly affects the expression of cell cycle-, cell apoptosis-, and oxidative stress-related proteins in the c-kit^+^ cells from irradiated mice. These results suggest that HW consumption is able to protect against TBI-induced HSC injury.

## 2. Materials and Methods

### 2.1. Mice

Male C57BL/6 mice were purchased from the Institute of Laboratory Animal Sciences, Chinese Academy of Medical Sciences & Peking Union Medical College. Mice were bred in the experimental animal center of the Institute of Radiation Medicine. Mice were used at approximately 6–8 weeks of age. All of the animal experiments in our study were approved by the Animal Care and Ethics Committee at the Institute of Radiation Medicine. The study was performed in accordance with the principle of Institutional Animal Care and Ethics Committee guidelines.

### 2.2. Antibodies

The following antibodies were purchased from eBioscience (San Diego, CA, USA): anti-mouse CD3-APC (clone 145-2C11), anti-mouse CD117 (c-kit) APC (clone 2B8), anti-mouse Sca-1-PE (clone D7), anti-mouse CD34-FITC (clone RAM34), and anti-mouse Ki67-FITC (clone SolA15). The following antibodies were purchased from Biolegend (San Diego, CA, USA): Percp-conjugated streptavidin, anti-mouse B220-PE (clone RA3-6B2), anti-mouse CD11b-Percp (clone M1/70), anti-mouse Gr1-Percp (clone RB6-8C5), anti-mouse CD45.1-FITC (clone A20), anti-mouse CD45.2-PE (clone 104), biotin-conjugated anti-mouse Ter119 (clone TER119), anti-mouse B220 (clone RA3-6B2), anti-mouse Gr1 (clone RB6-8C5), anti-mouse CD11b (clone M1/70), anti-mouse CD4 (clone GK1.5), and anti-mouse CD8 (clone 53–6.7). The following antibodies were purchased from BD Bioscience (San Jose, CA, USA): anti-mouse annexin V-FITC and anti-mouse *γ*-H2AX-FITC. The following antibodies were purchased from Cell Signaling Technology (Danvers, MA, USA): anti-mouse NRF2, anti-mouse BCL-XL, and anti-mouse BAK. Anti-mouse p21 and anti-mouse *β*-actin were purchased from Abcam (Cambridge, England). Anti-mouse 8-oxoguanine was purchased from Millipore (Boston, MA, USA).

### 2.3. Preparation of HW

The preparation of HW was based on a previous study with slight modification. H2 gas was generated from a hydrogen gas generator (SHC-300, Saikesaisi HW Energy, Shandong, China) and bubbled into 500 mL of sterile water at a rate of 150 mL/min for 20 min. The concentration of H_2_ in the water was detected with a dissolved hydrogen meter (Trustlex ENH-1000, Japan). HW was freshly prepared each day to ensure that an H_2_ concentration of more than 0.8 ppm was maintained.

### 2.4. TBI and HW Administration

Mice were divided into four groups for a 30-day survival experiment; the groups included 6.8 Gy TBI + vehicle (normal water), 6.8 Gy TBI + HW, 7.2 Gy TBI + vehicle, and 7.2 Gy TBI + HW. For the other experiments, mice were also divided into 4 groups, namely, vehicle, HW alone, 4 Gy TBI, and TBI + HW. The mice with TBI were irradiated with *γ*-rays at a dosage rate of 0.99 Gy/min. Mice received 0.5 mL HW by gavage for 10 min before TBI and up to the 7 days following TBI. The control mice with vehicle received normal water for the same frequency and volume as those in the mice with HW. Mice were finally euthanized on the 15th day after TBI.

### 2.5. Peripheral Blood Cell and Bone Marrow Cell Counts

Blood was obtained from mice via the orbital sinus and collected in micropipettes coated with EDTA. K_3_, white blood cells (WBCs), the percentages of lymphocytes (LY%), and neutrophil granulocytes (NE%) were counted and calculated. Bone marrow cells were isolated from tibias and femurs as previously reported [16], and cell numbers were analyzed with a hematology analyzer (Nihon Kohden, Japan).

### 2.6. Flow Cytometry Analysis

Bone marrow cells were suspended in phosphate-buffered saline (PBS), and cells were filtered and counted prior to antibody staining. To analyze the B cells, T cells, and myeloid cells in peripheral blood, 50 *μ*L of peripheral blood was harvested and stained with B220, CD3, CD11b, and Gr1 antibodies at room temperature. The red blood cells in the blood samples were removed using the BD FACS™ lysing solution. For HSC analysis, 5 × 10^6^ bone marrow cells were first stained with biotin-labeled Ter119, B220, Gr1, CD11b, CD4, and CD8 antibodies and subsequently stained with streptavidin, c-kit, sca1, and CD34 antibodies. To assess ROS levels, 1 × 10^6^ bone marrow cells were stained with anti-c-kit antibody and then incubated with 2,7-dichlorodihydrofluorescein diacetate (DCFDA, Beyotime Biotechnology, Nanjing, China, 10 *μ*M), MitoSox (Life Technologies, Grand Island, NY, USA; 10 *μ*M), and dihydroethidium (DHE, Beyotime Biotechnology, Nanjing, China, 5 *μ*M) in a 37°C water bath. For cell cycle analysis, bone marrow cells were first stained with a c-kit antibody and fixed and permeabilized with Cytofix/Cytoperm buffer (BD Biosciences, USA) before being stained with anti-Ki67 antibody and PI. For analysis of *γ*-H2AX, NRF2, HO-1, NQO1, BCL-XL, and BAK, bone marrow cells were first processed as described for ki67 staining and then staining *γ*-H2AX, NRF2, HO-1, NQO1, BCL-XL, and BAK antibodies at room temperature. For apoptosis analysis, bone marrow cells were also stained with c-kit and annexin V and PI according to the instructions for a BD apoptosis kit. Data acquisition was conducted on a BD Accuri C6 and analyzed using BD Accuri C6 software (BD Bioscience, San Jose, CA, USA).

### 2.7. Colony-Forming Units of Granulocyte Macrophage Cells (CFU-GM) Assay

A total of 1 × 10^4^ bone marrow cells in the nonirradiated control mice or 1 × 10^5^ bone marrow cells in the 4-Gy TBI mice were cultured in M3534 methylcellulose medium (Stem Cell Technologies, Vancouver, Canada) for 5 days. CFU-GM colonies with more than 30 cells were counted according to the kit instructions. The results were expressed as the number of CFU-GM per 10^5^ bone marrow cells.

### 2.8. Competitive Transplantation Assay

For competitive bone marrow cell transplantation, 1 × 10^6^ bone marrow cells from C57BL/6(CD45.2) mice and 1 × 10^6^ competitive cells from C57BL/6(CD45.1/45.2) mice were transplanted into lethally irradiated C57BL/6 mice (CD45.1). The percentage of donor-derived (CD45.2 positive) cells in the recipients' peripheral blood was examined 4 months after transplantation.

### 2.9. Isolation of c-kit^+^ Cells

Bone marrow cells were stained with c-kit-APC antibody for 30 min on ice, washed with PBS, and resuspended the pellet with anti-APC microbeads (Miltenyi Biotec, Germany) for 15 min. c-kit^+^ cells were sorted with a LS column in the magnetic separation filed. For analysis of ROS level and DNA damage, 1 × 10^6^ c-kit positive cells were cultured in 1 mL hydrogen-rich Stem Span® serum-free expansion medium (SFEM) 10 min before irradiation at 4 Gy. The cells were harvested for *γ*-H2AX and ROS analysis 0.5 hours and 18 hours after irradiation, respectively. For analysis of ROS level, SFEM medium (both hydrogen-rich medium and normal medium) renewed half of the liquid volume every 3 hours.

### 2.10. Protein Analyses

C-kit^+^ cells were lysed in ice-cold lysis buffer (BOSTER, Wuhan, China) containing 1 mM phenylmethanesulfonyl fluoride (PMSF). Western blot analysis was performed with anti-p21 (1 : 1000) and anti-GAPDH (1 : 5000).

### 2.11. Immunofluorescence Staining

C-kit positive cells were sorted as described above and fixed in 4% paraformaldehyde for 10 mins at room temperature. Cells were permeabilized in 0.2% Triton-X-100/PBS and blocked with 5% BSA for 1 h at room temperature and then incubated with anti-*γ*-H2AX (1 : 400) and 8-oxoguanine antibodies (1 : 300) overnight at 4°C and incubated with FITC-conjugated goat anti-rabbit IgG (1 : 300) and goat anti-mouse IgG (1 : 300) antibody for 1 h at room temperature.

### 2.12. Analysis of Hydroxyl Radicals (^∙^OH) and of SOD and GPX Enzyme Activities

Hydroxyl radicals and SOD and GPX enzyme activity were detected using detection kits (Nanjing Jiancheng Bioengineering Institute, Nanjing, China; and Beyotime Biotechnology, Nanjing, China) according to the manufacturer's instructions. A Fenton reaction occurred in the process of ^∙^OH detection following the manufacturer's instruction, and absorbance value was measured after chromogenic reaction happened. Level of ^∙^OH was evaluated and calculated by the formula following the manufacturer's instruction:

(1) Ability  of  OH•  production=OD  value T−COD  value S−B×8.824×1sample  volume×dilution  ratio.

B is the blank tube, S is the standard tube, C is the control tube, and T is the testing tube.

### 2.13. Statistical Analysis

Data are shown as means ± SEM, and an unpaired *t*-test (two-tails) was used for the majority of comparisons, along with Welch's correction *t*-test when the variances were not equal. Comparisons of overall survival were performed using log-rank test. Statistical analyses were performed using GraphPad Prism 5 software. A *P* < 0.05 represented statistical significance.

## 3. Results

### 3.1. HW Improves the Survival of Lethally Irradiated Mice

**Figure 1:**
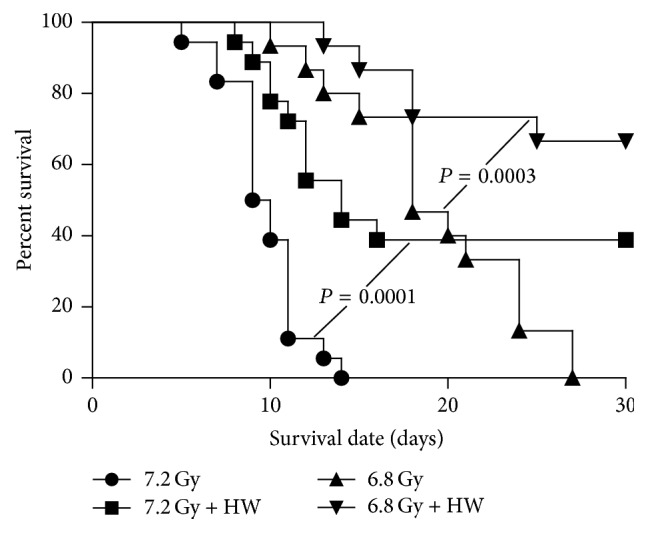
HW elevates the 30-day survival rate of mice receiving 6.8 Gy and 7.2 Gy TBI. Mice received 0.5 mL of vehicle water or HW administrated intragastrically 10 min before TBI and for 7 days after TBI. Curve chart shows the 30-day survival rate after exposure to a lethal dose of TBI. *N* = 15 in 6.8 Gy and 6.8 Gy + HW; *N* = 18 in 7.2 Gy and 7.2 Gy + HW.

To test whether HW affected the survival of mice after TBI, we fed mice with 0.5 mL of HW 10 min before 6.8 Gy or 7.2 Gy TBI and then kept HW consumption daily for 7 days after irradiation. As shown in Figure 1, all mice irradiated at 6.8 Gy or 7.2 Gy died within 27 days or 15 days following TBI. However, approximately 67% of mice exposed to 6.8 Gy and 40% of mice exposed to 7.2 Gy were alive 30 days after TBI under HW consumption. These findings suggest that HW significantly increases the survival of irradiated mice, at least 6.8 Gy and 7.2 Gy.

### 3.2. HW Alleviates Myelosuppression and Promotes Myeloid Skewing Recovery in Irradiated Mice

**Figure 2:**
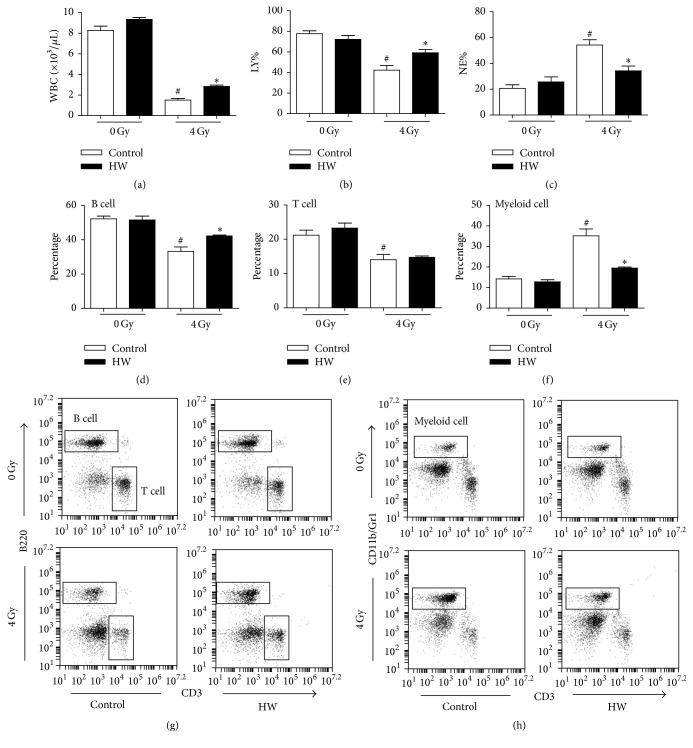
HW alleviates TBI-induced differentiation dysfunction in the hematopoietic system. (a) The bar graph shows the number of WBCs in peripheral blood. (b) The bar graph shows the percentage of lymphocytes (LY) in peripheral blood. (c) The bar graph shows the percentage of neutrophilic granulocytes (NE) in peripheral blood. (d) The bar graph shows the percentage of B cells in peripheral blood, as detected by FACS. (e) The bar graph shows the percentage of T cells in peripheral blood, as detected by FACS. (f) The bar graph shows the percentage of myeloid cells in peripheral blood, as detected by FACS. (g) Representative FACS analysis showing the percentage of B cells and T cells. (h) Representative FACS analysis showing the percentage of myeloid cells. All the data represent the mean ± SEM (*n* = 5); ^#^*P* < 0.05 versus 0 Gy control; ^*∗*^*P* < 0.05 versus 4 Gy control.

It has been well established that TBI can induce myelosuppression, a condition in which bone marrow activity decreased, resulting in a significant decline of peripheral blood cells [17, 18]. Wang and colleagues showed that lymphoid-biased HSCs were more sensitive to radiation-induced differentiation than myeloid-biased HSCs, resulting in myeloid skewing in irradiated mice [19]. Thus, to determine if HW consumption affected radiation-caused myelosuppression, we analyzed the number alteration of peripheral blood cells and the percentages of B cells, T cells, and myeloid cells. As illustrated in Figure 2, the irradiated mice exposed to 4 Gy TBI exhibited a significant decrease of WBCs and lymphocyte percentage (LY%) in peripheral blood 15 days following irradiation compared to the unirradiated controls. Moreover, the percentages of B cells and T cells, as detected by flow cytometry, were also declined. Conversely, there was an increase in both neutrophilic granulocyte percentage (NE%) and myeloid cell number in irradiated mice compared to unirradiated mice (Figures 2(c) and 2(f)). These findings indicated that TBI could result in myelosuppression and myeloid skewing. Irradiated mice with HW uptaken showed an increase of WBC counts, LY%, and B cell percentages and a decrease of NE% and myeloid cell percentage in the peripheral blood (Figures 2(c) and 2(f)). No alteration of T cell numbers was found in mice with TBI + HW. These results suggest that HW consumption improves mice recovery from TBI-induced myelosuppression and myeloid skewing.

### 3.3. HW Increases Number of Bone Marrow Cells (BMCs) of Irradiated Mice

**Figure 3:**
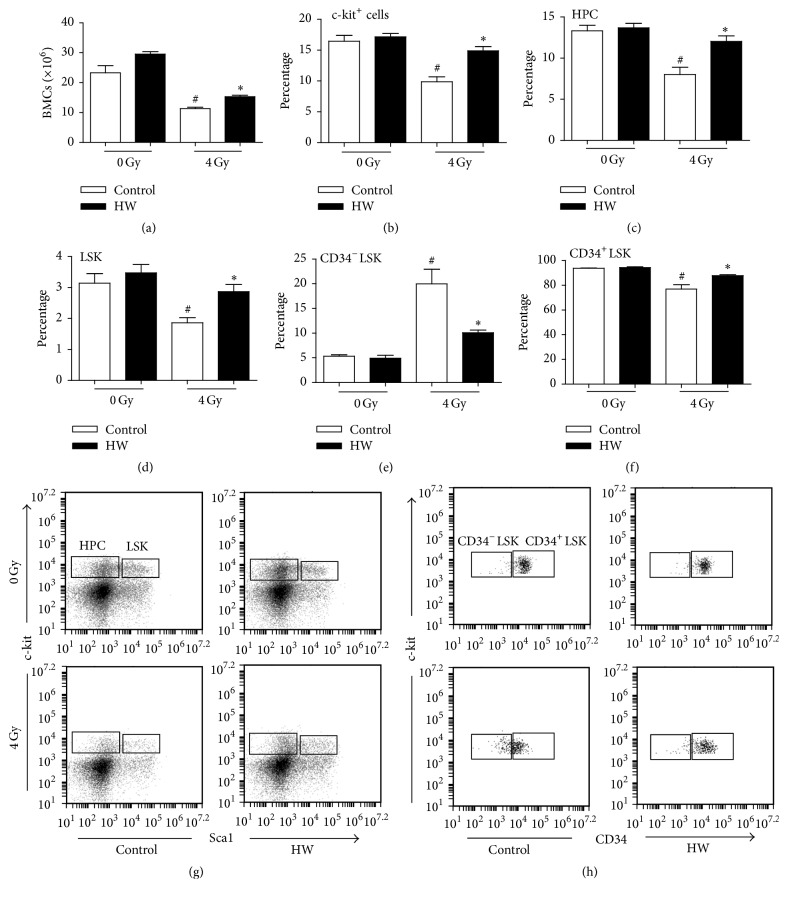
HW attenuates the TBI-induced decrease in BMCs, hematopoietic stem cells, and progenitor cells. (a) The bar graph shows the number of BMCs per femur. (b) The bar graph shows the percentage of c-kit^+^ cells among lineage-negative cells. (c) The bar graph shows the percentage of HPCs (Lineage^−^sca1^−^c-kit^+^BMCs) among lineage-negative cells. (d) The bar graph shows the percentage of LSKs (Lineage^−^sca1^+^c-kit^+^BMCs) among lineage-negative cells. (e) The bar graph shows the percentage of CD34^−^LSKs among LSK cells. (f) The bar graph shows the percentage of CD34^+^LSKs among LSK cells. The data are presented as the mean ± SEM of cell numbers per femur (*n* = 6). (g) Representative FACS analysis of the percentage of LSKs and HPCs. (h) Representative analysis of the percentage of CD34^−^LSKs and CD34^+^LSKs. All the data represent the mean ± SEM (*n* = 5); ^#^*P* < 0.05 versus 0 Gy control; ^*∗*^*P* < 0.05 versus 4 Gy control.

To determine whether HW consumption affected BMCs, we analyzed number alteration of BMCs per femur and the percentages of c-kit^+^ cells (Lineage^−^c-kit^+^BMCs), HPCs (Lineage^−^sca1^−^c-kit^+^BMCs), LSKs (Lineage^−^sca1^+^c-kit^+^BMCs), CD34^−^LSK, and CD34^+^LSK cells. As shown in Figure 3, 4 Gy TBI caused a decreased number of BMCs, a decrease of c-kit^+^ cells, HPCs, and LSKs, CD34^+^LSK frequency, and an increase of CD34^−^LSK percentage in mice at day 15 after irradiation compared to unirradiated mice. However, HW consumption reduced or inhibited these effects caused by TBI, that is, BMCs (Figure 3(a)), c-kit^+^ cells (Figure 3(b)), HPCs (Figure 3(c)), LSKs (Figure 3(d)), CD34^−^LSK frequency (Figure 3(e)), and CD34^+^LSK frequency (Figure 3(f)) in mice with TBI. These results suggested that HW could also improve mice recovery from TBI-induced alterations in BMCs.

### 3.4. HW Increases BMC Self-Renewal Ability in Irradiated Mice

**Figure 4:**
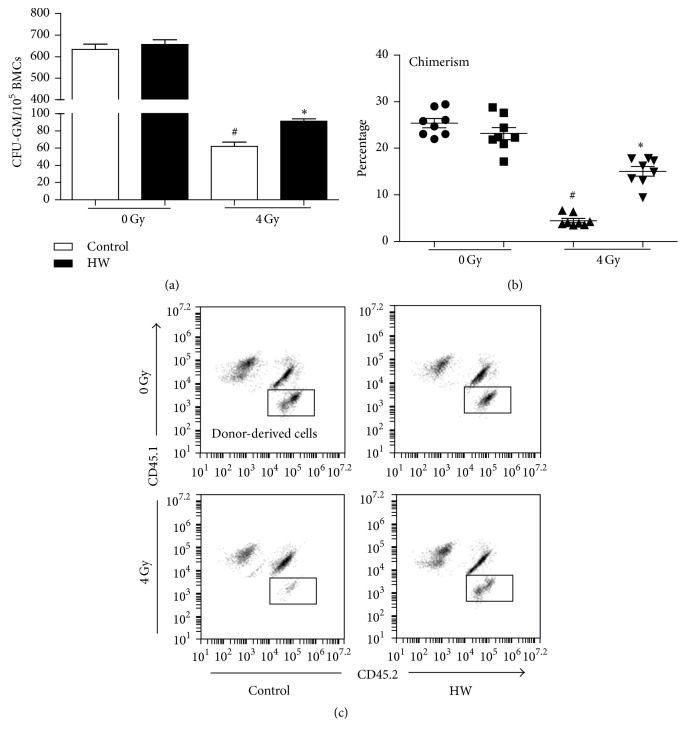
HW attenuates the TBI-induced decrease in self-renewal in HPCs and HSCs. (a) The bar graph shows the number of CFU-GM per 10^5^ BMCs. (b) The scatter plot shows the percentage of donor-derived cells in peripheral blood cells 4 months after transplantation. (c) Representative FACS analysis of the donor cell engraftment. All the data represent the mean ± SEM (*n* = 6 in panels (a); *n* = 8 in panel (b)); ^#^*P* < 0.05 versus 0 Gy control; ^*∗*^*P* < 0.05 versus 4 Gy control.

TBI causes an injury in self-renewal ability of HSCs [20, 21]. 4 Gy TBI significantly decreased CFU-GM number compared to the unirradiated mice: HW rescued such decline (Figure 4(a)). The competitive bone marrow transplantation assay is a gold standard for evaluating the self-renewal and regeneration capability of HSC [16]. Thus, we also transplanted lethally irradiated (CD45.1^+^) recipient mice with bone marrow cells from unirradiated donor mice or from donor mice with 4 Gy or 4 Gy + HW. The donor-derived cells (CD45.2^+^) in the peripheral blood of recipient mice were measured by FACS 16 weeks after transplantation. The chimerism of donor cells in irradiated mice (about 4%) was significantly lower than that in unirradiated mice (about 25%), while the donor cells in mice with 4 Gy + HW showed increased chimerism (about 15%) (Figures 4(b) and 4(c)). These results suggested that HW consumption could improve BMC self-renewal ability of irradiated mice.

### 3.5. HW Decreases ^∙^OH Levels in the c-kit^+^ Cells of Irradiated Mice

**Figure 5:**
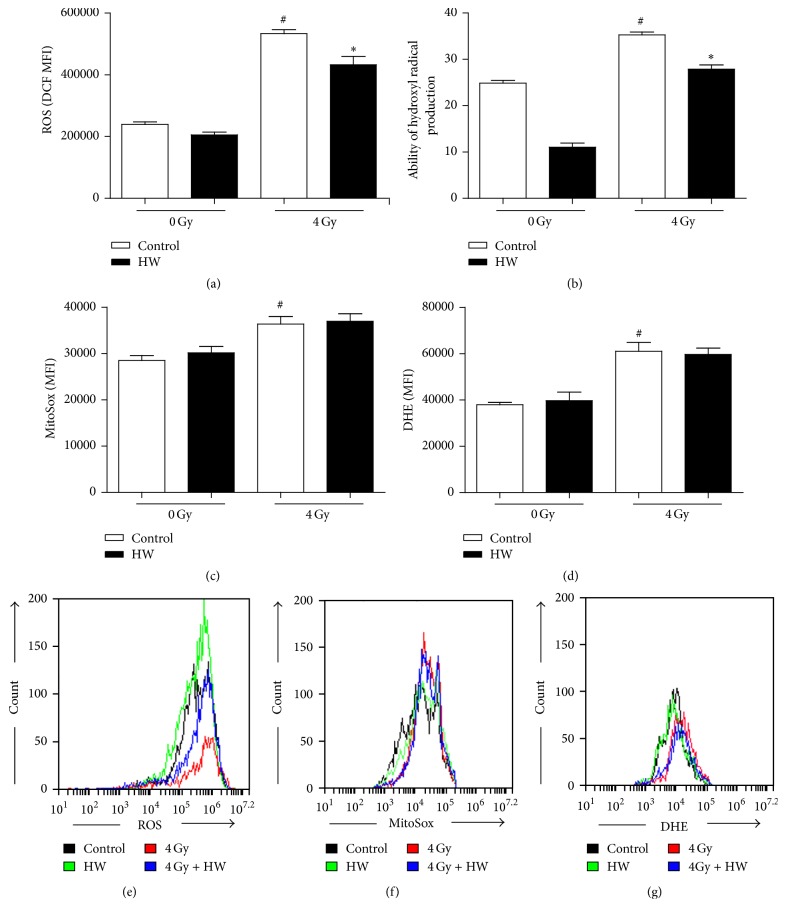
HW decreases TBI-induced ROS production in c-kit^+^ cells. (a) The bar graph shows the levels of intracellular ROS, as detected by DCF mean fluorescence intensity (MFI). (b) The bar graph shows hydroxyl radical production, as detected by the Fenton reaction. (c) The bar graph shows the levels of intracellular ROS, as detected by DHE MFI. (d) The bar graph shows the intracellular ROS in mitochondria, as detected by the MitoSox MFI. (e–g) Representative FACS plots of the ROS levels detected by DCF, DHE, and MitoSox MFI. All the data represent the mean ± SEM (*n* = 5 in panels (a), (c), and (d); *n* = 3 in panel (b)); ^#^*P* < 0.05 versus 0 Gy control; ^*∗*^*P* < 0.05 versus 4 Gy control.

To determine whether HW consumption affected ROS levels in irradiated c-kit^+^ cells, we treated c-kit^+^ cells with 10 *μ*M DCFDA, 10 *μ*M MitoSox, and 5 *μ*M DHE to detect total cellular ROS, mitochondria-derived ROS (superoxide), and superoxide free radicals, respectively. The ^∙^OH level was measured with a detection kit and evaluated by the ability to produce ^∙^OH as described in the Materials and Methods. All of three ROS types increased in c-kit^+^ cells of mice with 4 Gy TBI in comparison to unirradiated mice, while HW consumption decreased the total ROS (Figures 5(a) and 5(e)) and ^∙^OH levels (Figure 5(b)) and showed no effects on the mitochondria-derived ROS levels (Figures 5(c) and 5(f)) or the superoxide free radical levels (Figures 5(d) and 5(g)) in irradiated mice. These results indicated that HW consumption decreases ^∙^OH levels in c-kit^+^ cells of irradiated mouse.

### 3.6. HW Affects Cell Cycle and Cell Apoptosis in c-kit^+^ Cells of Irradiated Mice

**Figure 6:**
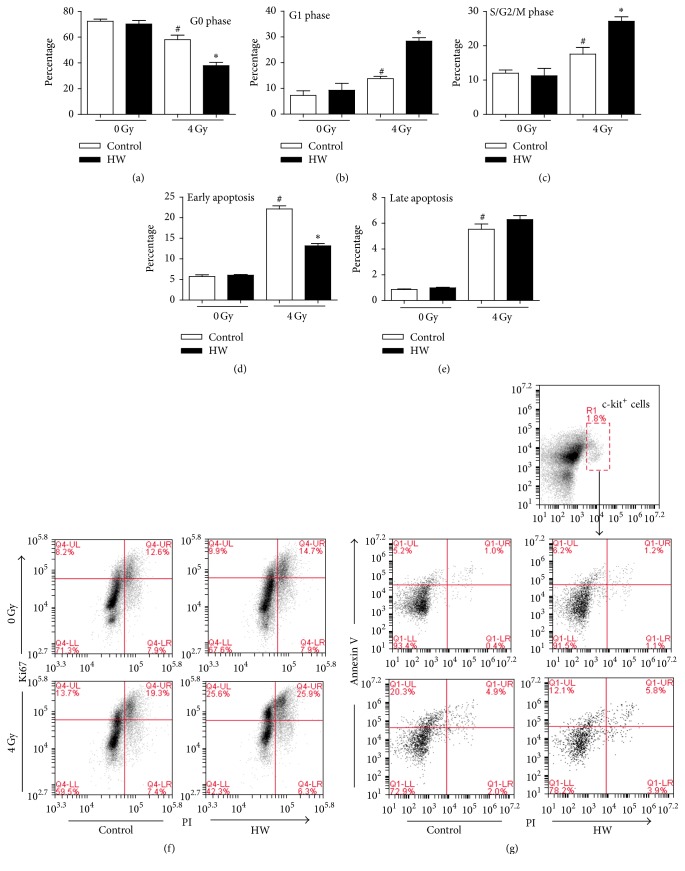
HW increases proliferation and decreases apoptosis in irradiated c-kit^+^ cells. (a) The bar graph shows the percentage of G0 phase (ki67^−^PI^−^) cells among BMCs. (b) The bar graph shows the percentage of G1 phase (ki67^+^PI^−^) cells among BMCs. (c) The bar graph shows the percentage of S/G2/M phase (ki67^+^PI^+^) cells among BMCs. (d) The bar graph shows the percentage of early apoptotic cells (annexin V^+^PI^−^). (e) The bar graph shows the percentage of late apoptotic cells (annexin V^+^PI^+^). (f) Representative FACS analysis of cell cycle status in BMCs. (g) Representative FACS plots of apoptosis in c-kit^+^ cells. All data represent the mean ± SEM (*n* = 4). ^#^*P* < 0.05 versus 0 Gy control; ^*∗*^*P* < 0.05 versus 4 Gy control.

As shown in Figure 6, 4 Gy TBI caused a significant arrest at G1-phase cells (about 28%) and S/G2/M phase (about 27%) of c-kit^+^ cells (about 38%) after 15 days in mice receiving a TBI at 4 Gy. Apoptosis (early and late apoptosis) was also observed to be increased in irradiated c-kit^+^ cells after irradiation. As expected, HW rescued TBI-induced cell cycle arrest and decreased the percentage of early apoptosis. These results showed that HW consumption indeed affected TBI-mediated cell cycle progression and early apoptosis of c-kit^+^ cells in irradiated mice.

### 3.7. HW Decreases DNA Damage and Oxidative DNA Damage in c-kit^+^ Cells of Irradiated Mice

**Figure 7:**
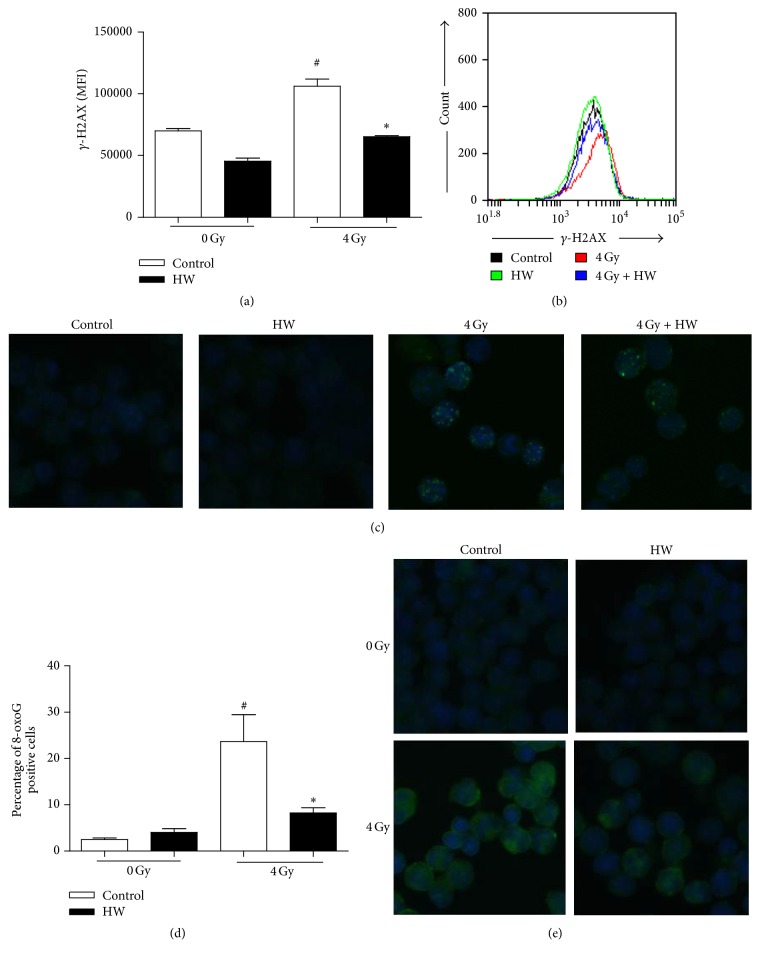
HW decreases DNA damage and oxidative DNA damage in irradiated c-kit^+^ cells. (a) The bar graph shows MFI of *γ*-H2AX in c-kit^+^ cells, as detected by flow cytometry. (b) Representative FACS plots of *γ*-H2AX. (c) Representative images of *γ*-H2AX in c-kit^+^ cells, as detected by immunofluorescence (IF). (d) The bar graph shows the percentage of 8-oxoG positive cells in c-kit^+^ cells. (e) Representative images of 8-oxoG in c-kit^+^ cells, as detected by IF. All the data represent the mean ± SEM (*n* = 5 in panel (a) and *n* = 3 in panel (d)); ^#^*P* < 0.05 versus 0 Gy control; ^*∗*^*P* < 0.05 versus 4 Gy control.

As reported previously, TBI can induce persistent oxidative stress in HSCs which may cause sustained DNA damage and oxidative DNA damage [22]. 8-oxoguanine (8-oxoG) is a naturally abundant base and impactful oxidative DNA lesions with a well-characterized mutagenic potential [23]; it is easily generated in DNA by reactive oxygen species induced by TBI [24]. Our results showed that MFI of *γ*-H2AX and percentage of 8-oxoG positive cells increased significantly in irradiated c-kit^+^ cells compared to control group, and HW consumption downregulated expression of *γ*-H2AX and 8-oxoG (Figure 7). These results showed that HW consumption decreased DNA damage and oxidative DNA damage in c-kit^+^ cells 15 days after 4 Gy TBI.

### 3.8. HW Upregulates Expression Antioxidation Proteins

**Figure 8:**
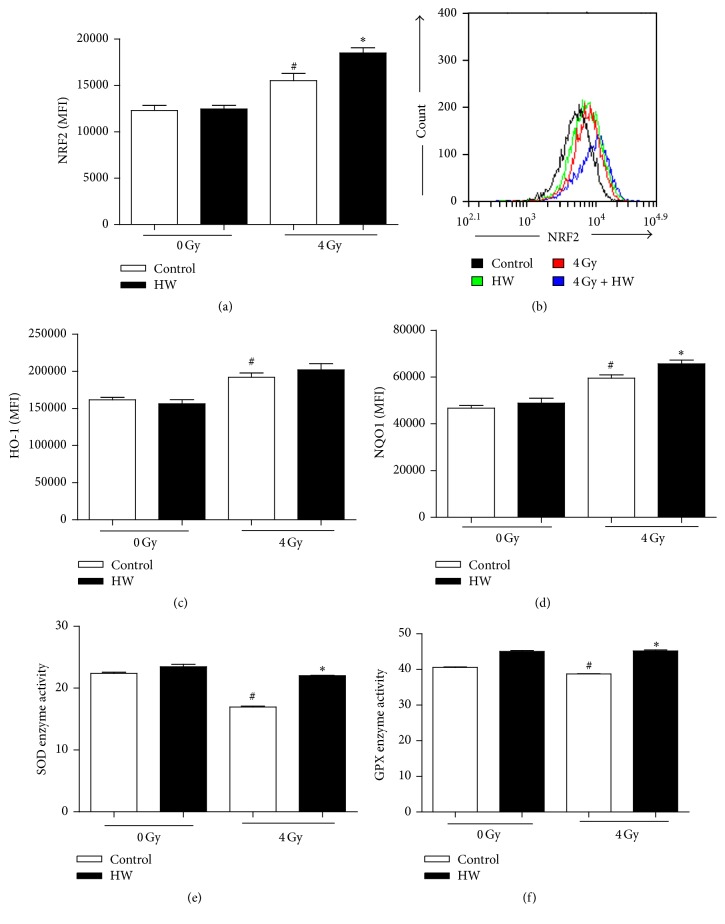
HW upregulates expression of NRF2 targeted proteins and enzyme activity of SOD2 and GPX1 in mice c-kit^+^ cells 15 days after 4 Gy TBI. (a) Bar graphs show the protein level of NRF2 in c-kit^+^ cells, as detected by FACS. (b) Representative FACS plots of NRF2. (c-d) Bar graphs show the protein level HO-1 and NQO1 in c-kit^+^ cells, as detected by FACS. (e-f) Bar graphs show the total enzyme activities of SOD and GPX in c-kit^+^ cells. All the data represent the mean ± SEM (*n* = 5 in panels (a), (c), and (d) and *n* = 3 in panels (e)-(f)); ^#^*P* < 0.05 versus 0 Gy control; ^*∗*^*P* < 0.05 versus 4 Gy control.

Nuclear factor erythroid 2-related factor 2 (NRF2) is a cellular sensor of oxidative stress [25]. In response to oxidative stress, NRF2 dissociates from kelch like ECH-associated protein 1 (KEAP1) and translocates into the nucleus which in turns regulates the transcription of heme oxygenase-1(HO-1) and NAD (P)H: quinine oxidoreductase 1 (NQO1) [26, 27]. Moreover, NRF2 is found to promote the survival of irradiated cells, including BM cells, as a result of ROS scavenging [28]. To explore whether HW consumption affects expression antioxidation proteins, we detected expression of NRF2, HO-1, and NQO1 by FACS and SOD, GPX1 enzyme activity. As shown in Figure 8, HW consumption upregulated expression of NRF2 targeted proteins and enzyme activity of SOD and GPX1. These results illustrated that HW decreases ROS level through upregulating expression antioxidation proteins 15 days after 4 Gy TBI.

### 3.9. HW Affects the Expression of Proteins Related to Proliferation and Apoptosis

**Figure 9:**
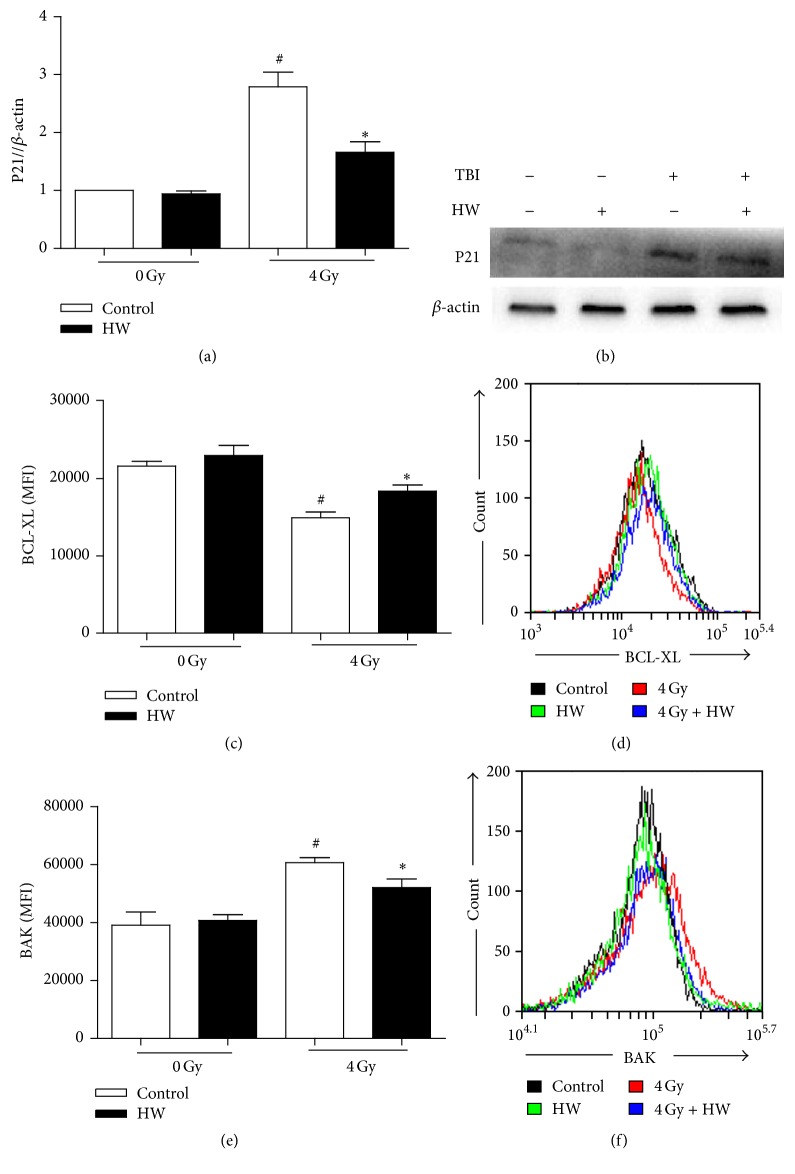
Protein levels of P21, BCL-XL, and BAK in mice c-kit^+^ cells 15 days after 4 Gy TBI. (a) Bar graph shows the relative expression of P21 protein level. (b) Representative western blotting analysis of the P21 in c-kit^+^ cells. (c) Bar graph shows the protein levels of BCL-XL as detected by FACS. (d) Representative FACS plots of BCL-XL protein levels. (e) Bar graph shows the protein levels of BAK as detected by FACS. (f) Representative FACS plots of BAK protein levels. Values represent the mean ± SEM (*n* = 3 in panel (a) and *n* = 5 in panels (c) and (e)). ^#^*P* < 0.05 versus 0 Gy control; ^*∗*^*P* < 0.05 versus 4 Gy control.

As shown in Figure 9, a downregulation of P21 (also known as cdkn1a or Cip1), a cell cycle inhibitor, is also involved in cell apoptosis and transcriptional regulation after DNA damage [29–31] (Figures 9(a) and 9(b)) and BAK, a proapoptotic protein, were observed at protein levels in HW-treated irradiated mice c-kit^+^ cells (Figures 9(e) and 9(f)). In contrast, the protein levels of the antiapoptotic protein BCL-XL were upregulated in c-kit^+^ cells of mice bearing TBI + HW (Figures 9(c) and 9(d)). These results revealed the alteration of these proteins expressions may be potential mechanisms involved in HW radioprotection in c-kit cells.

### 3.10. HW Decreases ROS Level and DNA Damage in c-kit^+^ Cells with 4 Gy IR In Vitro

**Figure 10:**
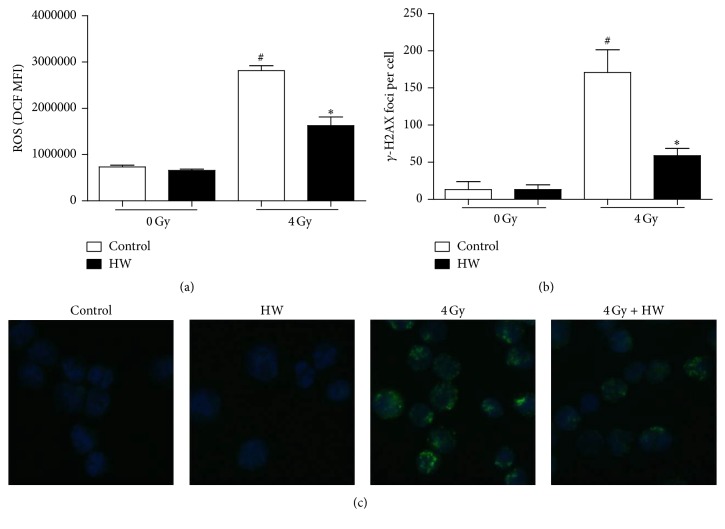
HW decreases ROS level and DNA damage in c-kit^+^ cells exposed to 4 Gy IR in vitro. (a) Bar graph shows the level of intracellular ROS 18 hours after 4 Gy IR in vitro. (b) Bar graph shows the number of *γ*-H2AX foci per cell 0.5 hours after 4 Gy IR in vitro. (c) Representative images of *γ*-H2AX in c-kit^+^ cells, as detected by IF. All the data represent the mean ± SEM (*n* = 3); ^#^*P* < 0.05 versus 0 Gy control; ^*∗*^*P* < 0.05 versus 4 Gy control.

To explore the direct radioprotective effect of HW, c-kit^+^ cells were cultured with hydrogen-rich SFEM medium 10 min before 4 Gy IR, and then we detected ROS level and DNA damage 18 hours and 0.5 hours after IR, respectively. As shown in Figure 10, when cultured with hydrogen-rich medium, DCF MFI and *γ*-H2AX foci in irradiated c-kit positive cells decreased significantly compared to cells cultured with normal medium. These results suggested that HW alleviated ROS production and DNA damage in vitro.

## 4. Discussion

It has been reported that H_2_ protects mice from TBI-induced bone marrow injury; however, existing research in the literature has focused on cultured hematopoietic cells or WBCs in peripheral blood, colony-forming units in the spleen, or the number of bone marrow cells in mice. The effect of H_2_ on TBI-induced HSC injury and its underlying mechanisms are poorly understood.

Guo et al. [32] and Chuai et al. [6] reported that mice with HW consumption 5 min before TBI alleviated radiation-induced hematopoietic system injury. Liu and colleagues reported that the hydrogen concentrations in the blood and tissues reached a maximum at 5 minutes after the oral administration of HW and then decreased slowly within 30 mins [33]. It has been widely accepted that with oral intake drugs or any other agents, drugs were absorbed into blood through gastrointestinal venous and then transported to all the tissues and organs in the body. In the publication of Liu et al., with oral administration of HW with concentration of 1.25 ppm, H2 blood levels did not increase up to an administered dose of 2.5 ppm; however, H2 levels in liver, kidney, heart, spleen, pancreas, intestine, muscle, and brain increased significantly when rats were treated with 1.25 ppm. So it is probably that H2 increase in bone marrow cells after mice were treated with 1.25 ppm HW (concentration of H2 in our experiment is about 1 ppm). Based on those previous studies, we administrated HW to mice 10 min (exactly, 5–10 min each mice per group) before TBI and 7 days after TBI. To confirm whether H2 reached bone marrow 10 mins after oral administration of HW, we measured ROS level in bone marrow cells after 4 Gy TBI (data not shown). In brief, mice were divided into 4 groups, control, 4 Gy, 4 Gy + amifostine, and 4 Gy + HW, 4 mice per group, and two independent experiments were executed. Amifostine is a ROS scavenger and radioprotective drug that has been approved by the US Food and Drug Administration (FDA), and mice received 200 mg/kg amifostine 30 mins before TBI in 4 Gy + amifostine group and 0.5 mL HW 10 mins before TBI in 4 Gy + HW group. Mice were sacrificed immediately after 4 Gy TBI and bone marrow cells were harvested; then we measured ROS level with flow cytometry. When treating irradiated mice with amifostine or HW, ROS decreased significantly which show indirect evidence that H2 reach to bone marrow 10 mins after HW consumption.

Our results show that the number of WBCs in peripheral blood and the number of BMCs per femur 15 days after irradiation are increased when irradiated mice are supplied with HW. These results are consistent with other studies [6]. Our present findings demonstrate that HW consumption can counteract imbalances in myeloid-lymphoid differentiation, increase the percentages of c-kit^+^ cells, LSKs, HPCs, and CD34^+^LSKs, and decrease the CD34^−^LSKs frequency in TBI mice. Notably, self-renewal and reconstitution were significantly improved by HW.

To explore the underlying mechanisms, we measured the ROS levels, cell proliferation, cell apoptosis, DNA damage, oxidative DNA damage, and the expression of related proteins. HW consumption decreases total ROS level and selectively reduces the ^∙^OH level in irradiated c-kit^+^ cells. In contrast, no significant alteration of mitochondria-derived ROS (superoxide) and superoxide free radicals were observed between c-kit^+^ cells with TBI and c-kit^+^ cells with TBI + HW. These data confirm that H_2_ selectively reduces ^∙^OH level, this result is consistent with the results obtained by other researchers [3, 34]. In addition, HW results in an increased percentage of proliferative cells, a decreased proportion of early apoptotic cells and the frequency of DNA damage, and oxidative DNA damage in irradiated c-kit^+^ cells by regulating related proteins. Consistent with the experiments in vivo, when culturing c-kit^+^ cells with hydrogen-rich SFEM medium in vitro, ROS level and *γ*-H2AX foci decreased significantly compared to these cells cultured with normal SFEM medium. The variation in cell proliferation, cell apoptosis, and DNA damage may contribute to the increased cell numbers and frequencies observed in HW-treated BMCs.

It has been reported that ROS in bone marrow cells increased significantly at 7, 9, 15, 22, 30, and 56 days after mice received TBI [22, 35–39]. However, it is a very short period that ROS persist after TBI, how should these radicals be generated 15 days after TBI? In our experiments, there are almost no LSKs and HPCs 4 h after TBI, then survival HSCs replenish themselves and differentiate into blood cells of all hematopoietic lineages, LSKs and HPCs appear about 10 days after TBI with high level ROS production, and the reason why ROS is produced in newly generated hematopoietic stem cells deserved to be further explored. According to the published research, NRF2 and targeted genes and proteins play an important role in ROS production in irradiated hematopoietic stem cell [28]. Our unpublished works focus on the effect of the systemic (circulatory) environment on TBI-induced bone marrow cells injury, indicating that components in irradiated mice circulatory environment may regulate expression of NRF2 and targeted genes and proteins. So next we detected expression of antioxidative proteins in c-kit^+^ cells.

H_2_ has been reported to elevate the production of SOD and GSH in the thymus [40] and testis [41]. Consistent with these studies, we demonstrate that H_2_ enhances the activity of SOD and GPX enzyme. A number of in vitro and in vivo experiments have found that H_2_ as a potent antioxidant activates NRF2 and its downstream signaling molecules [42–49]. Our results show that H_2_ elevates the expression of NRF2 and targeted protein (HO-1 and NQO1) in c-kit^+^ cells of TBI mice. However, the mechanism by which H_2_ activates* NRF2* and its related signaling pathways is unknown and warrants further exploration. In addition, our results show that H_2_ upregulates the expression of the antiapoptotic protein BCL-XL, consistently with prior studies [9]. H_2_ downregulates the expression of the cell cycle inhibitor P21 and the proapoptotic protein BAK in irradiated c-kit^+^ cells. These findings suggest that H_2_ may regulate many other signaling pathways to protect BMCs from TBI-induced injury.

Taken together, our present study preliminarily reveals the protective effect of H_2_ by HW consumption on TBI-induced HSC injury with alteration of the related proteins. Future studies are carried on to further study the molecule mechanism by which H_2_ mediated radioprotection of HSCs. H_2_, as a low-toxicity agent, shows promising radioprotective effects on TBI-induced injury in many organs and tissues. Future studies are needed to determine how to counteract issues related to solubility and stability.
